# Buccal mucosal graft onlay repair for a ureteric ischemic injury following a pyeloplasty

**DOI:** 10.4103/0970-1591.60458

**Published:** 2010

**Authors:** Vipul Agrawal, Vimal Dassi, Mukund G. Andankar

**Affiliations:** Department of Urology, BYL Nair Hospital, Mumbai, India

**Keywords:** Omentum pedicle, onlay buccal mucosa patch graft, ureteric strictures

## Abstract

A 38-year-old female presented with long stricture in the left upper ureter following a pyeloplasty causing persistent flank pain. A left PCNL with an antegrade endopyelotomy was attempted in view of a concomitant left renal 1.5 cm calculus in the lower calyx but it failed. Subsequently, a buccal mucosal onlay graft was applied on the strictured ureter. Follow-up at 3 months showed good uptake of the graft with patent passage for urine drainage.

## INTRODUCTION

Stricture in the upper ureter is commonly seen following surgical procedures of the upper urinary tract and ischemic injury is one of the important causative factors in such cases. A multitude of procedures have been described for repair of ureteric strictures. Complicated ureteric strictures of the upper ureter may require bowel replacement or autotransplantation. However, both these procedures are associated with long-term morbidity and complications. Successful use of a buccal mucosa graft in repair of a stricture in the urethra has led to interest in the use of similar grafts in repair of complicated ureteric strictures, though only two series of five cases each have been reported.

## CASE REPORT

We report a case of stricture ureter following laparoscopic pyeloplasty, which was successfully repaired using an onlay buccal mucosa patch graft.

The patient, a 38-year-old married female attended our out patient department (OPD) with a history of left flank pain. She had a history of bilateral flank pain, for which she had undergone the following:

1. Multiple sessions of extra corporeal shockwave lithotripsy (ESWL) - for bilateral renal calculi (twice on the right side and five times on the left side from April 2004 to January 2006).
2. Right laparoscopic pyeloplasty performed on 10, August 2006.
3. Left laparoscopic pyeloplasty performed on September 2007; the stone could not be retrieved.

**Figure 1:**
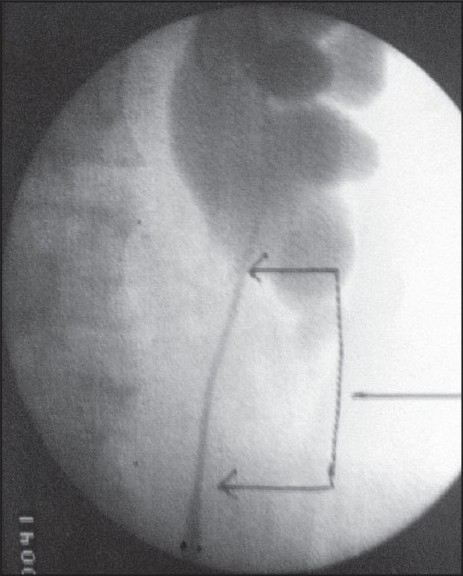
Left retrograde pyelography after an antegrade endopyelotomy

The left flank pain recurred after 2 months of the stent was removed. Her retrograde pyelography showed a renal calculus with a long upper ureteric stricture. In view of the concomitant renal calculus, a left percutaneous nephrolithotripsy (PCNL) with antegrade endopyelotomy was peformed in 2008 but the pain persisted upon stent removal. A repeat retrograde pyelography showed worsening of the stricture [Figure 1].

Thus, an open repair of the strictured ureter using buccal mucosal onlay was performed on August 11, 2008. Per-op findings showed the stricturous segment to be 7 cm long. A graft was harvested from the right inner cheek and sutured to the stricturous segment with Vicryl 4/0 over a 7/14 Fr endopyelotomy stent. A omentum pedicle was wrapped around the graft [Figure 2]. A follow-up retrograde pyelography and intravenous urography showed excellent graft uptake, with no contrast leak, good patency of the repaired segment, and normal renal function [Figure 3].

**Figure 2a:**
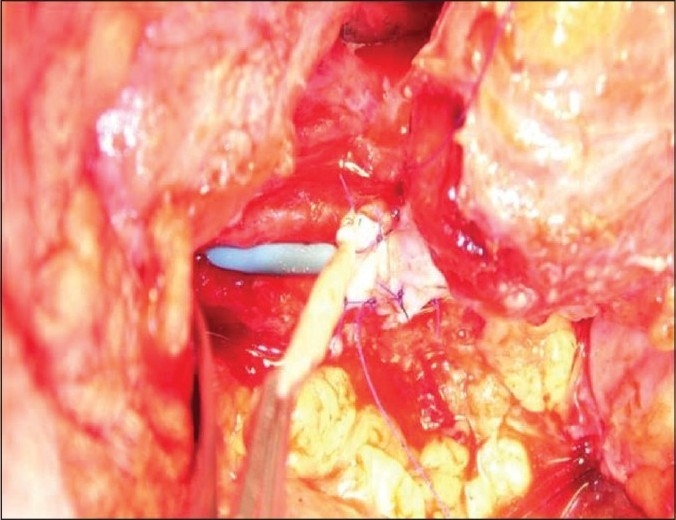
Suturing of an onlay graft

**Figure 2b:**
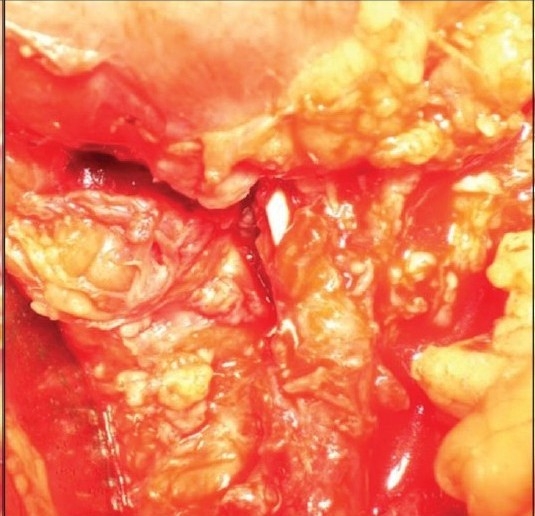
Suturing of a graft over a stent with omental pedicle

**Figure 3:**
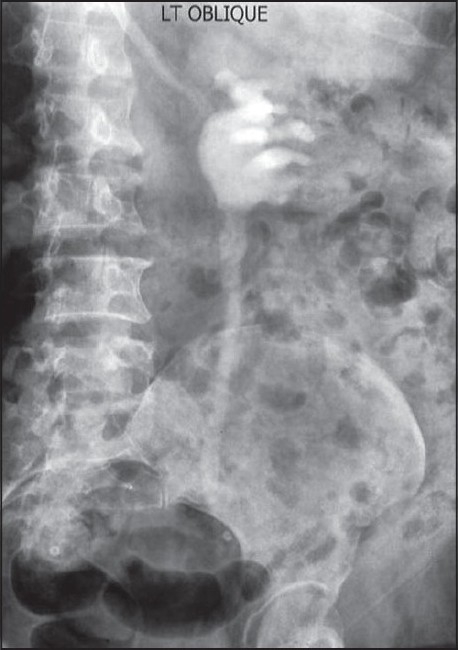
Follow-up intravenous pyelography at 3 months

## DISCUSSION

Stricture in the upper ureter is commonly seen following surgical procedures of the upper urinary tract and ischemic injury is one of the important causative factors in such cases. Complicated ureteric strictures and segmental ureteric loss beyond the anticipated reach of a Boari flap might warrant bowel interposition or auto transplantation. Both procedures are of considerable surgical magnitude and entail long-term complications.

The classic Davis intubated ureterotomy (1943)[1] requires prolonged stenting and success depends on the damaged urothelium to regenerate.

Ureteral reconstruction has been performed using an autologous bladder mucosa graft[2] and free flaps of parietal peritoneum.[3] However, due to disruption, these materials did not come into vogue. It has been confirmed often that an adynamic ureteric segment, if patent, allows free drainage of the upper tract.

Successful use of a buccal mucosa graft in repair of hypospadias and a strictured urethra has led to interest in the use of similar grafts in repair of complicated ureteric strictures. However, until now there has been limited data on the success of this procedure.

In experimental animals, buccal mucosa interposition grafts have been shown to take well and to provide good drainage of the upper tract. Somerville and Naude[4] performed an animal study using a tubularised, free, buccal mucosal graft for segmental ureteric replacement and found excellent take-up of the graft and upper tract drainage.

Ureteric strictures too long to be treated by excision and spatulated end-to-end anastamosis can be effectively treated by buccal mucosal patch graft, which may be easily harvested from the cheek. The graft ensures patency and good drainage and avoids more radical alternatives such as bowel interposition or autotransplantation. Buccal mucosal grafts demonstrate an abundant layer of vasculature in the outer layer of the lamina propria. In addition, the excellent vascular bed is provided by omental wrap. Grafts to the ureter have an excellent take-up rate. Another advantage is that there is no need to depend on the severely damaged urothelium to regenerate, with minimal chances of urinary leakage postoperatively.

Buccal mucosa can be used as a patch graft or a tubularised graft. Ureteral reconstruction by dorsal onlay graft technique is simple and devoid of complications. It ensures patency and good drainage.

Following his success in animal models, Naude[5] reported the successful use of buccal mucosal patch grafts in 5 patients having a variety of ureteric lesions, and a tubularised buccal mucosal graft in 1 patient with a segmental ureteric loss. The patients had a variety of ureteric lesions due to tuberculosis, bilharziasis, severe fibrosis, and stenosis of ureteropelvic junction (UPJ) following pyelolithotomy in the intrarenal pelvis, loss of a segment of the ureter following a gunshot injury, and resection of a peri-ureteric mass. Ureteric patency was established and maintained in all patients. There were no complications and urine was sterile in all patients at follow-up. Thus, buccal mucosal patch graft repair proved capable of maintaining patency and good urinary drainage in patients with complicated ureteric strictures and segmental ureteric loss.

Shah, *et al*.,[6] reported a ureteroplasty using buccal mucosal onlay grafts with omental wrap in 5 patients with long, benign ureteric strictures due to tuberculosis in 4 patients and amyloidosis in 1 patient. In all the patients, the length of the stricture was greater than 5 cm. Ureteric patency was established in all patients. Four patients had persistent improvement in renal function. In 1 patient, renal function improved initially (at 6 months follow-up) but subsequently (at 1-year follow-up) the unit was non functioning.

Follow-up was in the range of 1.5 year to 3.5 years. Thus, the graft proved capable of maintaining patency and good urinary drainage. The procedure is technically simple and devoid of complications.
